# A mechanobiological computer optimization framework to design scaffolds to enhance bone regeneration

**DOI:** 10.3389/fbioe.2022.980727

**Published:** 2022-09-07

**Authors:** Camille Perier-Metz, Georg N. Duda, Sara Checa

**Affiliations:** ^1^ Berlin Institute of Health at Charité, Julius Wolff Institute, Universitätsmedizin Berlin, Berlin, Germany; ^2^ MINES ParisTech, PSL Research University, Paris, France

**Keywords:** bone scaffold, bone regeneration, scaffold design optimization, computational mechanobiology, surrogate optimization

## Abstract

The treatment of large bone defects is a clinical challenge. 3D printed scaffolds are a promising treatment option for such critical-size defects. However, the design of scaffolds to treat such defects is challenging due to the large number of variables impacting bone regeneration; material stiffness, architecture or equivalent scaffold stiffness—due it specific architecture—have all been demonstrated to impact cell behavior and regeneration outcome. Computer design optimization is a powerful tool to find optimal design solutions within a large parameter space for given anatomical constraints. Following this approach, scaffold structures have been optimized to avoid mechanical failure while providing beneficial mechanical stimulation for bone formation within the scaffold pores immediately after implantation. However, due to the dynamics of the bone regeneration process, the mechanical conditions do change from immediately after surgery throughout healing, thus influencing the regeneration process. Therefore, we propose a computer framework to optimize scaffold designs that allows to promote the final bone regeneration outcome. The framework combines a previously developed and validated mechanobiological bone regeneration computer model, a surrogate model for bone healing outcome and an optimization algorithm to optimize scaffold design based on the level of regenerated bone volume. The capability of the framework is verified by optimization of a cylindrical scaffold for the treatment of a critical-size tibia defect, using a clinically relevant large animal model. The combined framework allowed to predict the long-term healing outcome. Such novel approach opens up new opportunities for sustainable strategies in scaffold designs of bone regeneration.

## 1 Introduction

Large bone defects do not heal spontaneously if they exceed a critical dimension. The current gold standard treatment in such clinical cases consists of autologous bone grafting but has several drawbacks such as the need for a second surgery and a limited amount of adequate bone graft tissue (Dimitriou et al., 2011; Schlundt et al., 2018). 3D-printed scaffolds appear as a promising alternative treatment strategy for large bone defects, with good pre-clinical results (Reichert et al., 2012; Pobloth et al., 2018). However, their translation to the clinic has been limited so far, partly due to the large number of scaffold design variables influencing the regeneration process, which limits the predictability of the clinical outcome; hence, bone scaffold design is often the result of a trial-and-error process.

Several research groups have departed from a trial-and-error approach by using computational optimization methods when designing bone scaffolds. A common approach consisted in maximizing or minimizing specific mechanical properties such as stiffness to mimic the material properties of bone tissue (Almeida and da Silva Bártolo, 2010; Chen et al., 2011; Xiao et al., 2012; Dias et al., 2014; Wang et al., 2016; Metz et al., 2020). Such concept may be considered limited, since endogenous bone regeneration is limited in too stiff environments (Prendergast et al., 1997; Pobloth et al., 2018). Other groups have targeted specific values for stiffness and/or diffusivity of the scaffold to mimic the tissue being replaced (Hollister et al., 2002; Hollister and Lin, 2007; Sturm et al., 2010; Wieding et al., 2014; Makowski and Kuś, 2016; Chang et al., 2017). Nonetheless, scaffold properties mimicking a missing tissue do not ensure being supportive to the endogenous bone regeneration process (Petersen et al., 2018).

Few studies have focused on the mechanobiological potential of a scaffold design as a means of optimization. Boccaccio and colleagues optimized periodic scaffolds to provide favorable mechanical conditions for bone regeneration immediately after implantation (Boccaccio et al., 2016a, 2016b, 2018a, 2018b; Percoco et al., 2020). However, such approach is also limited as the bone regeneration process is known to be highly dynamic, where initial optimal conditions cannot ensure an optimal regeneration outcome (Perier-Metz et al., 2021). Over the course of bone healing, different types of tissues are formed, remodeled and resorbed in specific areas of a defect, creating a highly dynamic local mechanical niche that changes over time and further influences cell response and tissue formation. A few studies confirmed that designs optimized for the situation immediately after surgery did not yield optimal bone regeneration in various set-ups (Bashkuev et al., 2015; Perier-Metz et al., 2021; Wu et al., 2021). These findings suggest that bone scaffold optimization should rather be performed on an intended regeneration outcome and not only the initial post-surgery setting, preferably as a time-dependent mechanobiological optimization (Metz et al., 2020).

So far, to the best of our knowledge, only two time-dependent, mechanobiology-based bone scaffold optimization studies have been performed. In the first one, Poh et al. (2019) optimized a scaffold porosity distribution for a large defect in a sheep long bone, based on a simplified bone regeneration model. However, their model did not include any other tissue type than bone (e.g., cartilage, fibrous tissue). Furthermore, the optimization set-up was restricted to 1D, meaning that the actual scaffold micro-structure was not optimized. More recently, Wu and colleagues proposed a method for a time-dependent mechanobiology-based topology optimization of bone scaffolds (Wu et al., 2021). However, also this model did not account for any other tissue type but bone nor for the needed initial cell infiltration into the defect.

To overcome these limitations, we propose a novel *in silico* framework to optimize scaffold design based on the level of regenerated bone volume aimed at the end of the regeneration process. Such prediction has to consider the activity of individual cells and the potential for different tissue types to be formed during the healing process. To achieve this, a computer framework combining a multiscale mechanobiological bone regeneration model, a surrogate model for bone healing outcome and an optimization algorithm was developed. This framework was applied to optimize scaffolds for a large defect in a sheep tibia.

## 2 Materials and methods

To realize an optimization taking into account the regeneration outcome, this study focuses on the design optimization of a cylindrical scaffold to be implanted in a large bone defect in the sheep tibia as an example. The *in silico* framework to optimize scaffolds was realized by using a finite element model that was coupled to an agent-based model to simulate bone regeneration within such scaffolds. This computer model was first used to simulate bone regeneration in a relatively large set of scaffold designs, to generate the interpolation data necessary for a surrogate model. Based on the results of the bone regeneration model, a simplified relationship between the scaffold design parameters and the predicted regenerated bone outcome was computed (i.e., surrogate model). Thereafter, this relationship was used in an optimization framework, to find the optimal scaffold design parameters to maximize the amount of regenerated bone.

### 2.1 Finite element model

**FIGURE 1:**
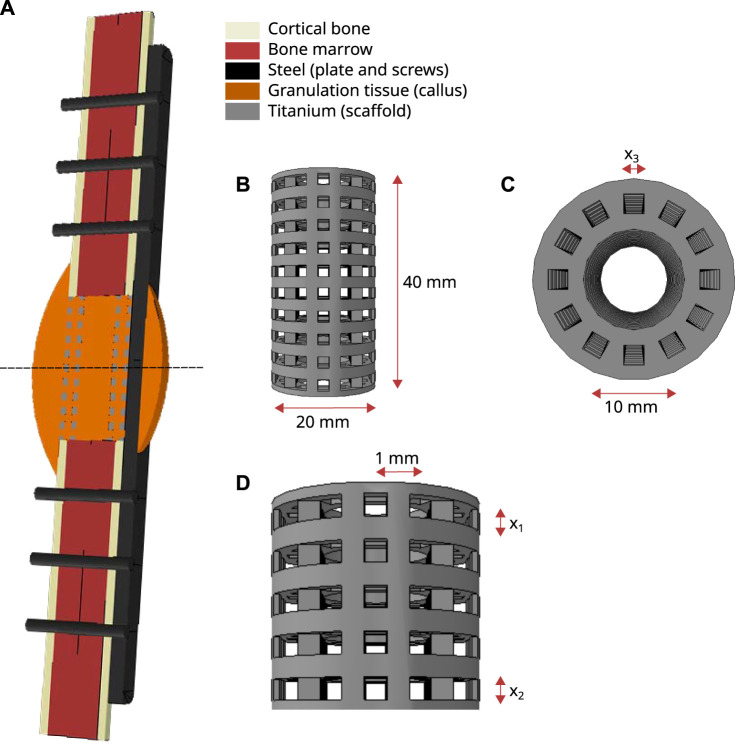
**(A)** Finite element model set-up: longitudinal cut through the defect configuration showing the intact bone extremities, the implanted scaffold and the fixation plate; the dashed line represents the symmetry plane used in the analysis. **(B–D)** Cylindrical scaffold description and parametrization: **(B)** longitudinal view of the full scaffold geometry; **(C)** radial view of the scaffold defining the vertical pore size parameter x_3_; **(D)** longitudinal view of the half scaffold (taking advantage of the symmetry) defining the horizontal pore size parameters x_1_ and x_2_.

A finite element model was developed where the geometry of the defect and the surrounding bone extremities was based on previously published *in silico-in vivo* studies (Pobloth et al., 2018; Perier-Metz et al., 2020). A 4-cm segmental defect in a sheep tibia was modelled by a cylinder of outer radius 10 mm (cortical bone) and inner radius 7.5 mm, containing the bone marrow (Figure 1A). The defect was modelled to be stabilized by an internal stainless steel fixation plate and six screws, following previous experimental setups (Pobloth et al., 2018). A regenerating zone, the callus, was modelled in and around the defect by rotating a circle arc of maximum width 10 mm at mid-height and overlapping the intact bone extremities by 10 mm.

A scaffold with outer radius 10 mm and inner radius 5 mm and pores with square sections following three perpendicular directions was modelled (Figures 1B,C). The pore centers had a spacing of 1 mm in both horizontal directions (longitudinal view, Figures 1B,D); whereas the 12 pores extruded along the vertical direction were placed regularly in a circle (radial view, Figure 1C). Three variables were defined to parametrize the scaffold geometry: x_1_ was the pore size closest to the bone extremities and x_2_ the pore size at mid-height of the scaffold, assuming a linear gradient of the pore sizes in the three rows between these (Figure 1D); and x_3_ defined the pore size in the radial plane (Figure 1C). The parameters x_1_ and x_2_ were constrained between 0.3 and 3.8 mm and x_3_ between 0.3 and 3 mm. The lower bound was dictated by biological requirements to ensure good vascularization and nutrient supply, as experimental studies suggested a minimal pore size of 300 µm (De Witte et al., 2018), while the upper bound was chosen to avoid that neighboring pores overlap.

This model was implemented in the finite element (FE) software Abaqus v.6.18 (Simulia, Rhode Island) to perform mechanical analyses. Because of the symmetry of the geometry, only one half of the model was simulated, following the dashed line along the horizontal plane at mid-height (Figure 1A). The different geometrical parts were meshed with second-order elements as follows: hexahedral elements of average size 2.5 mm for the intact cortical bone, the bone marrow and the plate; beam elements of size 1 mm for the screws; and tetrahedral elements of average sizes 1.2 mm for the callus and scaffold—this size was adapted depending on the size of the meshed geometry, in particular the scaffold struts.

The following constraints were defined between the different geometrical parts: the intact bone extremities (cortical bone and bone marrow) were tied to the callus; the screws were tied to the intact bone; the screw heads were constrained by multi-point constraints of type beam with the plate holes; the callus was constrained such that it was tied to the scaffold.

The biological parts (cortical bone and bone marrow) were modelled as poroelastic materials with properties summarized in Table 1. The callus space was assumed to be initially filled with granulation tissue (poroelastic material, Table 1). Over time, regenerating tissue material properties were updated according to the predicted tissue formation of the bone regeneration model (Section 2.2).

**TABLE 1:** Material properties (Perier-Metz et al., 2020).

Material	Young’s modulus (MPa)	Poisson’s ratio	Permeability (10^−14^ s.m^4^.N^−1^)	Bulk modulus grain (MPa)	Bulk modulus fluid (MPa)
Stainless steel	210,000	0.3	-	-	-
Titanium	104,000	0.3	-	-	-
Soft scaffold material	0.2	0.3	-	-	-
Cortical bone	17,000	0.3	0.001	13,920	2,300
Bone marrow	2	0.167	1	2,300	2,300
Granulation tissue	0.2	0.167	1	2,300	2,300
Fibrous tissue	2	0.167	1	2,300	2,300
Cartilage	10	0.3	0.5	3,700	2,300
Immature bone	1,000	0.3	10	13,940	2,300
Mature bone	17,000	0.3	37	13,940	2,300

A compression load of 1372 N and a bending moment of 17.125 Nm were applied on the proximal end of the bone, corresponding to two body weights (BW) proximal-distal compression and an anterior-posterior moment of 0.025 BWm at the fixed end of a 20-cm intact bone, respectively (Duda et al., 1997; Perier-Metz et al., 2020). A symmetry boundary condition was defined on the xy symmetry plane (“ZSYMM”). Lastly, pore pressure was constrained to be zero on the outer surfaces of the poroelastic parts (callus, cortical bone and bone marrow).

Two different scaffold optimization studies were performed, assuming different scaffold Young’s moduli (Table 1):

1) titanium, defined as a linear elastic material with Young’s modulus 104 GPa
2) a hypothetical very soft material, defined as a linear elastic material with Young’s modulus 0.2 MPa (similar to the Young’s modulus of granulation tissue)

### 2.2 Bone regeneration model

A previously described mechanobiological bone regeneration (MBBR) model, which was able to explain bone regeneration within scaffolds in different experimental setups, was used to predict tissue formation during the healing process (Perier-Metz et al., 2020, 2022). It consisted in a 3D agent-based computer model implemented in C++ and coupled with the afore-mentioned FE model (Section 2.1). Each agent, of size 100 μm, could represent one cell of one of the following phenotypes: progenitor cell, fibroblast, chondrocyte and immature or mature osteoblast, with the associated extracellular matrix: granulation tissue, fibrous tissue, cartilage and immature or mature bone, respectively.

Progenitor cells were allowed to migrate randomly with an average speed of 30 μm/h (Appeddu and Shur, 1994). The origin of these cells was mimicked by initially seeding them randomly in the bone marrow cavity and along the periosteum with a 30% occupancy rate (Perier-Metz et al., 2020). The rest of the tissue volume was considered cell-free at the initial time point.

Differentiation, proliferation and apoptosis of the cells depended on a mechanoregulation stimulus S based on octahedral shear strain γ and fluid velocity ν , defined by $S=\frac{\gamma }{a}+\frac{\nu }{b}$ , with the empirical parameters a and b: a=0.0375 and $b=0.03mm.s^{-1}$ (Huiskes et al., 1997; Lacroix and Prendergast, 2002). Mechanical thresholds were defined for the stimulus (S) favoring bone resorption (S < 0.01), mature bone (0.01 < S < 0.53), immature bone (0.53 < S < 1), cartilage (1 < S < 3) or fibrous tissue (S > 3) (Perier-Metz et al., 2020). Cells would proliferate with a predefined proliferation rate (60%, 55%, 20% or 30% for progenitor cells, fibroblasts, chondrocytes and osteoblasts, respectively) providing that they were in the right mechanoregulation stimulus; or would undergo apoptosis otherwise, with a different rate (5%, 5%, 10% or 16% for progenitor cells, fibroblasts, chondrocytes and osteoblasts, respectively) (Perier-Metz et al., 2020). Progenitor cells differentiated with a rate of 0.3 days^−1^ towards the cell phenotype corresponding to the local stimulus. In addition, surface-guided tissue deposition was implemented, meaning that differentiation could happen only following existing surfaces (tissues or scaffold), and graft was assumed to be present in the scaffold pores where it stimulated proliferation and differentiation, as previously described (Perier-Metz et al., 2020).

This agent-based model was coupled with the FE model in two ways: 1) the mechanoregulation stimulus that dictated the cell behaviors was derived element-wise from the FE analysis; and 2) the tissue material properties were updated in the callus of the FE model at every iteration depending on the agent-based model cell distribution. To do so, each element was mapped to the agents it contained and its material properties were defined according to a rule of mixtures: the average material properties of the tissues predicted in the agents within each single element was computed, and the obtained value was further averaged over the last ten iterations to account for the time needed for tissue deposition and maturation (Lacroix and Prendergast, 2002). The FE analysis and agent-based simulations were run iteratively to predict the full regeneration process, where one iteration represented one day.

### 2.3 Surrogate optimization set-up

**FIGURE 2:**
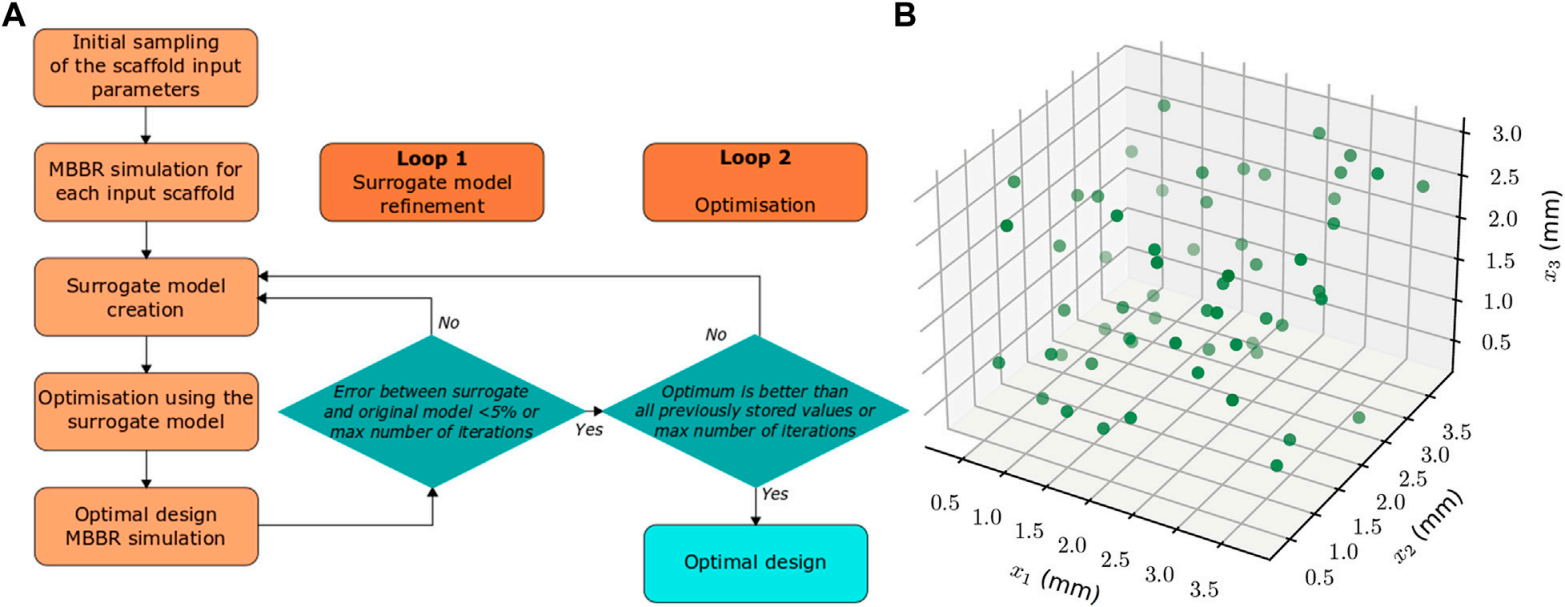
**(A)** Flow chart of the scaffold design optimization computational framework for enhanced bone regeneration. **(B)** Locations of the initial samples (in the space defined by the three parameters x_1_, x_2_, x_3_) for which the MBBR model is run to compute the surrogate model.

The bone regeneration model described above (Section 2.2) is computationally intensive (it takes around 6 h on a standard workstation to simulate one full regeneration process). However, for the optimization process, the regeneration outcome needs to be predicted multiple times, until the optimum scaffold is found. Therefore, to ensure that the optimization process runs in a reasonable time, a surrogate optimization approach was adopted. A surrogate computer model (a simplified input-output relationship) of the bone regeneration model described above (Section 2.2) was built based on a set of initially sampled input parameters obtained by a design of experiments technique. The input parameters were the scaffold design variables (x_1_-x_3_) whereas the output was defined as the predicted fraction of regenerated bone volume within the scaffold pores after 24 weeks. Thereafter, the scaffold design optimization was performed using the surrogate model. To ensure surrogate model accuracy and optimality of the outcome, the optimum design predicted by the surrogate model was computationally tested for bone regeneration outcome using both the surrogate model and the MBBR model. A first loop of the framework was used to ensure that the difference in the regenerated bone volume value between the surrogate and the MBBR model was below 5% (Figure 2A); as long as the error was bigger, the input values and the corresponding output (as simulated with the MBBR model) were added to the dataset to compute a new surrogate model, and a new optimum was determined based on this improved surrogate model. The second loop consisted in building the surrogate model with the given dataset and computing an optimum based on this surrogate model until an optimum was reached that was better than all previously stored values (to avoid local optima). The output was simulated again with the MBBR model to ensure a good confidence in the result. If a new loop was necessary, this new data point was added to the dataset to build the surrogate model and improve it further.

The initial dataset used to build the surrogate model was determined using the Latin Hypercube Sampling technique (Simon et al., 2002). Based on preliminary studies, 20 times the number of variables was found to be sufficient to obtain an accurate enough surrogate model, i.e, 60 samples in this set-up. The locations of the samples are given in Figure 2B.

The surrogate model was built using the predicted regenerated bone volume using the Matlab kriging toolbox DACE (Lophaven et al., 2002). An exponential auto-correlation was assumed as it gave the best results in a preliminary study. To perform the optimization, the Matlab Global optimization toolbox was used where different optimization algorithms were tested: direct search, genetic algorithm and particle swarm optimization (The MathWorks, Inc. 2020). For the direct search, an initial guess for x1, x2 and x3 has to be defined to initialize the optimization process: the values 2.05, 2.05, and 1.65 mm were arbitrarily used. In all algorithms, the default settings were used to perform the optimization.

In summary, the surrogate optimization framework includes the following steps (Figure 2A):

1) Run the MBBR model for a set of input parameters obtained by Latin Hypercube Sampling technique (LHS).
2) Build a kriging surrogate model based on the initial data using the Matlab kriging toolbox DACE.
3) Perform optimization based on the surrogate model predictions using the Matlab Global optimization toolbox (The MathWorks, Inc. 2020): direct search, genetic algorithm (GA), particle swarm optimization (PSO)
4) Run the MBBR model for the optimum scaffold parameters found in step 3 and compare the MBBR model output to the surrogate prediction (loop 1) or current optimal value (loop 2)
5) Repeat steps two to four to ensure model accuracy (loop 1, allowing max 5% discrepancy between the surrogate prediction and the MBBR model output) and optimality of the outcome (loop 2)

To automate the creation of the FE models for each scaffold design, a Python script was developed for Abaqus, which allowed to define the pore corner positions based on the pore size. MATLAB R2018b (The MathWorks, Inc. 2020) was used to run the actual optimization framework, including launching Abaqus CAE for the geometry update and the C++-Abaqus MBBR model. Pore size values (input) and corresponding output (bone volume in the scaffold pores) were stored in a text file for further analysis.

### 2.4 Analysis of the outcome

The main outcome of the MBBR simulations was the predicted regenerated bone volume fraction in the scaffold pores after 24 weeks. Regenerated bone volume fraction was computed as the total amount of agents within the scaffold pores occupied by osteoblasts divided by the total number of agent positions within the scaffold pores.

The porosity of the resulting scaffold was computed as the scaffold pore volume divided by the hollow cylinder volume (outer radius 10 mm and inner radius 5 mm). The regenerated bone volume fraction could then be related to the scaffold macroscopic porosity.

In addition, histology-like images of bone regeneration predictions were computed in the mid-sagittal plane of the defect using histology-like colors, similar to the experimental Safranin Orange/von Kossa staining: bone in black, fibrous tissue in light red and cartilage in dark red (Pobloth et al., 2018; Perier-Metz et al., 2020). This representation allowed a comparison between different designs and study cases.

## 3 Results

### 3.1 Titanium scaffold optimization

**FIGURE 3:**
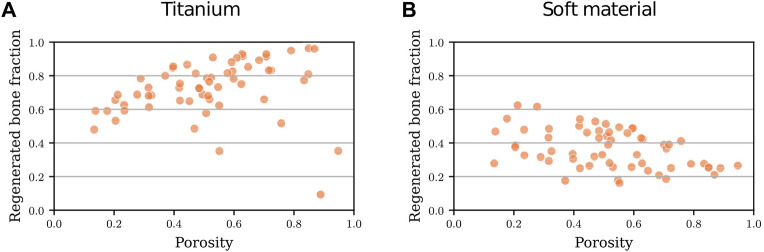
Predicted regenerated bone volume fraction (bone volume/total pore volume) within the scaffold pores for scaffold designs with different porosities for 60 initial scaffold designs: **(A)** made of titanium; **(B)** made of a soft material (Young’s modulus 0.2 MPa).

The first step of the optimization process of the titanium scaffold consisted in simulating the 60 initial designs defined by the Latin hypercube sampling strategy (Figure 2B).These simulations yielded a regenerated bone volume fraction in the scaffold pores varying from 10% to 95%. The corresponding designs had porosities ranging from 13% to 95%, with a tendency towards a better regeneration potential for higher porosities until ca. 90% (Figure 3A). Above this porosity, the designs yielded very bad healing outcomes due to high deformations stimulating fibrocartilage formation instead of bone. Nevertheless, the porosity alone was not a good indicator of the healing potential, as the same porosity yielded many different healing outcomes. For instance, 50%-porosity scaffolds yielded 35%–90% regenerated bone volume in the scaffold pores (Figure 3A).

**FIGURE 4:**
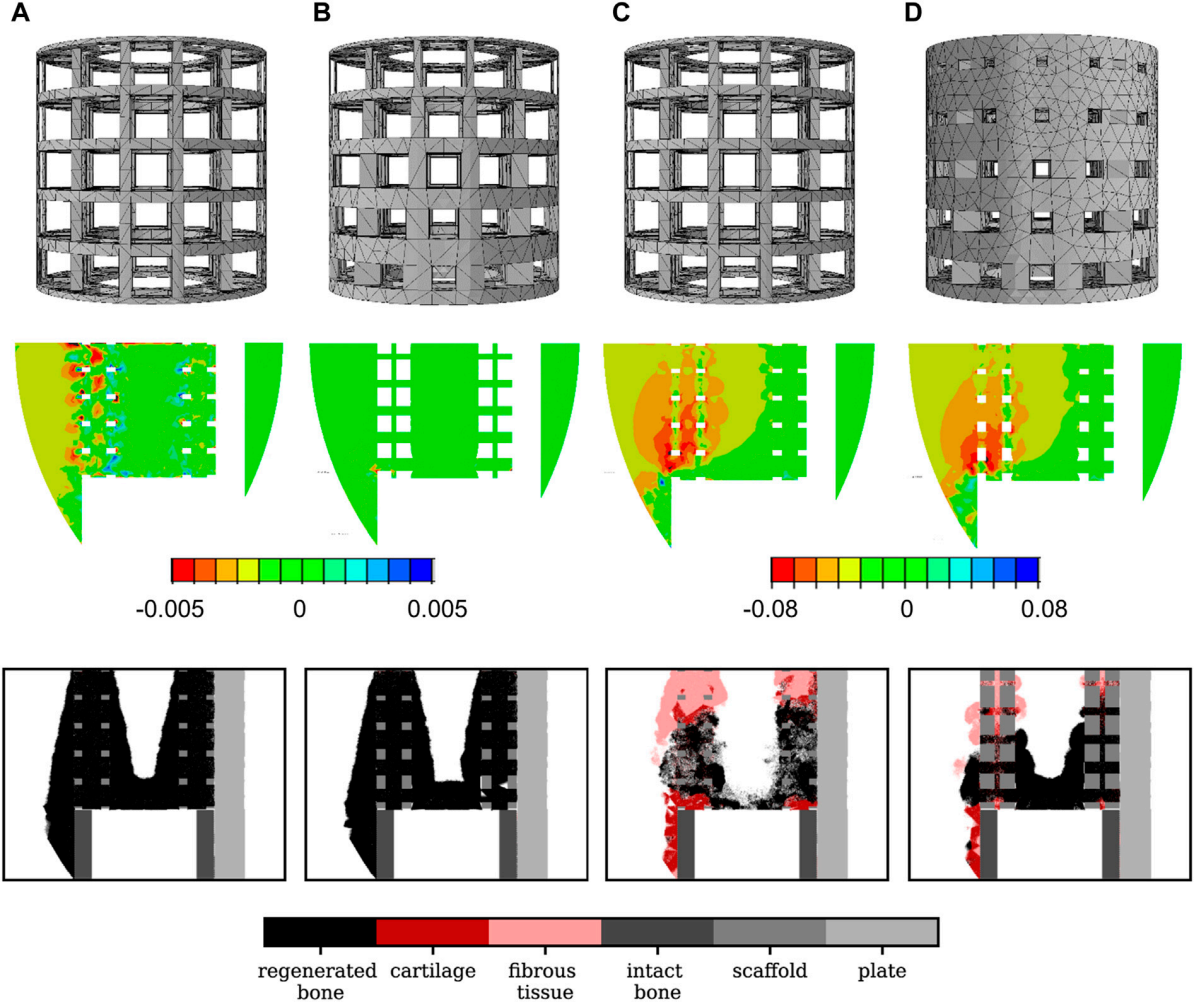
Scaffold design, initial absolute principal strain distribution in the mid-sagittal plane and 24-week histology predictions in the mid-sagittal plane for different scaffold designs: **(A)** optimal titanium scaffold design; **(B)** a good titanium graded design ( $X_{1}=1.84\mathrm{mm}$ $X_{2}=3.45\mathrm{mm}$ , $X_{3}=2.26\mathrm{mm}$ ); **(C)** scaffold design optimized for titanium but now made of soft material; **(D)** optimal soft material scaffold design.

The three tested optimization algorithms (direct search, GA and PSO) suggested similar optimal titanium scaffold designs yielding ca. 96% regenerated bone (Table 2). The best design was obtained with the PSO algorithm, where the scaffold design was defined by $x_{1}=3.12mm$ , $x_{2}=3.26mm$ and $x_{3}=2.6mm$ This scaffold had a porosity of 85% and yielded roughly 96% of regenerated bone in the scaffold pores (Figure 4A). The optimal scaffold did not show a clear advantage of a gradient in the scaffold pore size for bone regeneration, since the values of x_1_ and x_2_ were very close to each other. Interestingly, one of the very graded designs ( $x_{1}=1.84mm$ , $x_{2}=3.45mm$ , $x_{3}=2.26mm$ ), that was part of the interpolation dataset, performed nearly as well, with 93% regenerated bone but only 71% porosity (Figure 4B). Compared to the optimal design, some areas of bone resorption appeared around some scaffold walls.

**TABLE 2:** Optimization results for both scaffold compositions using various algorithms.

Scaffold material	Algorithm	$x_{1}$ (mm)	$x_{2}$ (mm)	$x_{3}$ (mm)	Porosity	Regenerated bone volume fraction
Titanium	Direct search	3.07	3.36	2.34	84%	96%
	GA	3.07	3.36	2.34	84%	96%
	PSO	3.12	3.26	2.64	85%	96%
Soft material	Direct search	1.62	1.44	0.98	29%	51%
	GA	1.9	0.50	0.70	22%	65%
	PSO	1.9	0.77	0.70	24%	67%

GA, genetic algorithm; PSO, particle swarm optimization.

### 3.2 Soft scaffold optimization

The predicted regenerated bone volume fractions for the interpolation dataset using soft material properties for the scaffold varied from 20% to 60%, thus showing a reduced healing potential compared to the titanium scaffolds. The corresponding designs of the interpolation dataset had the same geometries as the ones used for the titanium scaffold optimization; therefore, their porosities ranged from 13% to 95% as well. A tendency towards a lower regeneration potential for higher porosities was observed (Figure 3B). Also in this case, the porosity alone was not a good indicator of the healing potential, as e.g., 50%-porosity scaffolds yielded 15%–50% regenerated bone volume in the scaffold pores (Figure 3B).

The optimal titanium scaffold design was predicted to perform very badly when assuming that it would be made of the very soft material with Young’s modulus 0.2 MPa: large amounts of fibrous tissue and cartilage were predicted to grow. As a consequence, only 27% of bone was present in the scaffold pores after 24 weeks compared to 96% when the scaffold was made of titanium (Figure 4C). The much softer material yielded high strain values in the defect, thus leading to a mechanoregulation stimulus favoring more fibrocartilage formation.

The optimal soft scaffold was obtained with the PSO algorithm and the scaffold design was defined by $x_{1}=1.9mm$ , $x_{2}=0.77mm$ and $x_{3}=0.7mm$ (Table 2). This design was more graded than the titanium optimum (larger pores close to the intact bone extremities), but far less porous (24% porosity). It yielded only 67% regenerated bone in the scaffold pores after 24 weeks, but large amounts of fibrocartilage in the center of the defect (Figure 4D). The other two algorithms (direct search, GA) converged to different optima which were found to yield even less regenerated bone (51% and 65%, respectively) at similar levels of porosity (Table 2).

### 3.3 Computational efficiency

The optimization processes presented in Sections 3.1 and 3.2 took ca. 2 weeks to perform, including 10 days for the simulation of the bone regeneration process in the 60 initial scaffold geometries used to build the surrogate model and 2–7 days for each tested optimization algorithm (5–20 additional MBBR simulations) on a standard desktop PC. In particular, when using the PSO algorithm—the algorithm that performed best in both cases—for the optimization step, 100 h (ca. 4 days) of computation were needed for the titanium scaffold optimization and 26 h for the soft material scaffold, in addition to the initial 10 days for the initial dataset simulations. In comparison, a standard optimization approach would have required thousands of simulation runs lasting 6 h each, i.e., years of computations. Hence, the computing performance was improved by a factor of at least 100.

## 4 Discussion

We propose here a computational framework for time-dependent mechanobiological optimization of 3D-printed scaffolds towards enhanced bone regeneration. Our method is based on the bone regeneration outcome to optimize scaffold design, instead of only the post-surgery mechanical stimulus or scaffold mechanical properties. The framework uses surrogate modelling and design of experiments methods that allow for a set-up that can be run in a reasonable amount of time on standard computing machines. In this study, the framework was used to optimize a scaffold for the treatment of a large bone defect in sheep, where scaffolds of two different material properties were considered: titanium and a very soft material (with a Young’s modulus similar to that of granulation tissue).

The optimization of the titanium scaffold design suggested a very porous design with large pores (86% porosity) to be optimal for bone regeneration, yielding 96% regenerated bone in the scaffold pores. This result is in good agreement with an *in silico* study performed by Byrne and colleagues that found higher porosities to be more beneficial for bone regeneration (Byrne et al., 2007). A previous pre-clinical study has also shown that a honeycomb titanium scaffold with 84% porosity leads to bone regeneration within the here simulated large bone defect (Pobloth et al., 2018). For the experimental scaffold, the computer model of bone regeneration previously predicted a 98% regenerated bone in the scaffold pores (Perier-Metz et al., 2020). Even though the optimization was performed on a relatively simple scaffold geometry with square-section pores, the computed optimum proved capable of performing as good as a more complex experimental design. This first result is therefore very promising as the optimum of a simple parameterized geometry is already predicted to yield very good bone regeneration capabilities. Further studies should test the potential of the optimization approach in other, more complex scaffold designs, which could have a larger number of design parameters and therefore yield even better results.

A sensitivity analysis of the optimal titanium scaffold design to the loading conditions was further performed to assess the validity of the optimization process (Supplementary Material). Indeed, the optimal design was shown to still yield mostly bone regeneration under 50% higher or lower loading conditions, what suggested a good robustness of the design under loading uncertainties. Other bone scaffold optimization studies have shown that the optimal scaffold design (according to the initial mechanical stimulus) depended on the assumed loading conditions (Boccaccio et al., 2016a; 2016b; 2018a). Here, the lower loading conditions decreased more strongly the regenerated bone volume fraction within the scaffold pores (79% compared to 96%).

The optimization of the soft scaffold (Young’s modulus of 0.2 MPa) showed a very different result, with the optimum being far less porous (24%) and yielding less bone than the optimal titanium scaffold (67%). Interestingly, the design was graded, with larger pores close to the intact bone extremities and smaller ones in the center of the defect. This result is in line with a previous *in silico* study optimizing the porosity distribution of a large bone defect scaffold (Poh et al., 2019). A reason for this distribution of pore sizes might be the need to sustain higher strains in the center of the defect, as bone regenerates from the intact extremities towards the center so that the remaining granulation tissue in the center of the scaffold is compressed together from the bone formed above and below. Here, the chosen material was very soft; the experimental literature suggests intermediate material properties as an alternative to foster bone regeneration, e.g., polymers and/or ceramics (Reichert et al., 2012; Reznikov et al., 2019). Using our set-up, future studies could perform a polymer-ceramic composite scaffold optimization for large bone defect regeneration, including the degradable behavior of these materials.

Two previous studies have used mechanobiological algorithms to perform a time-dependent bone scaffold optimization: topology optimization studies in 2D (large defect) and 3D (partial defect) (Wu et al., 2021) and a porosity distribution optimization study in 1D (Poh et al., 2019). In the first case, the use of topology optimization techniques allowed a free design, where no topology was imposed *a priori*. However, Wu and colleagues did not take into account the actual cell invasion of the scaffold pores nor the different tissue types taking part in the bone regeneration process (e.g., fibrous tissue, cartilage). In addition, they assumed a periodic design and optimized only one (cubic or square) representative volume element of this scaffold, thus not allowing any variations between different scaffold regions (Wu et al., 2021). The second study consisted in a very fast method as it focused only on one dimension; however, it was strongly limited as the optimal scaffold micro-structure could therefore not be predicted (Poh et al., 2019). In fact, this method should be combined with a micro-structure optimization step for a practical usage by means of a multi-scale optimization (Dondl et al., 2019).

This study had some limitations related to the mechanobiological bone regeneration model. Vascularization was not included, although it is known to be a very limiting factor in large bone defect regeneration; however, this limitation was mitigated by the use of large pores to avoid impairing the re-vascularization process (minimum 300 µm) and the assumption that graft was implanted into the scaffold, which is likely to enhance the vascularization process. Moreover, the bone regeneration computer model used here has been shown to have good predictive capabilities in two independent experimental setups where different scaffolds designs were used (Perier-Metz et al., 2020, 2022). Another limitation was that each agent point was bigger than actual cell sizes (Isaksson et al., 2008), so that an agent was assumed to contain several cells and the corresponding tissue; tissue deposition was therefore not implemented as such. Preliminary studies showed that this simplification did not affect much the final regeneration predictions but rather their dynamics, what was not the focus of the present study. The relatively coarse mesh size is a further limitation of the MBBR model, which might in particular result in distorted predictions of the tissue composition and material properties due to the averaging of material properties over a full element; however, preliminary mesh convergence studies performed in a previous study showed that the mesh size did not impact the mechanoregulation stimulus prediction (Perier-Metz et al., 2020). In this study, optimization analyses were done for two specific scaffold materials, with the very stiff titanium on the one hand [comparable to (Pobloth et al., 2018)] and the very soft granulation tissue-like material on the other (Petersen et al., 2018); future studies should take into account degradable polymeric or composite (polymer-ceramic) materials that are considered for clinical usage. This would add a further dynamic aspect in the optimization process due to the degradation of the scaffold material. Also, it would have been possible to include the material stiffness in the optimization process, but this might be less realistic (only some stiffness values are possible) and would distort the optimization results, as the variation of material stiffness values can be of orders of magnitude compared to the pore size values. Lastly, despite the implementation of a surrogate modelling approach, the computation time (a few weeks for one scaffold design optimization set-up) is still too long for a clinical routine usage, where an optimal design should be designed in a few days for a patient-specific case. Future studies should take advantage of high-performance computing technologies by using dedicated hardware and parallelized algorithms to further reduce the computing time.

In summary, we propose here a technological platform that allows to optimize hierarchically-structured bone scaffold designs not only against mechanical failure and initial in-growth of bone but for a sustainable long-term optimized regenerative process. With improved computational efficiency and providing that the healing potential of an individual is known, this method could be employed for the development of personalized 3D-printed bone scaffolds to ensure an optimal regeneration outcome to a given patient.

## Data Availability

The raw data supporting the conclusion of this article will be made available by the authors, without undue reservation.
