# Supplementary material for: A mechanobiological computer optimization framework to design scaffolds to enhance bone regeneration
Source: Front Bioeng Biotechnol. 2022 Sep 7;10:980727. doi: 10.3389/fbioe.2022.980727 (PMC9490117; doi:10.3389/fbioe.2022.980727)
Supplement: Supplementary file 1 [file DataSheet1.docx]

Supplementary Material

# Sensitivity to the loading conditions

The MBBR simulations were run with changed loading conditions for the titanium optimal scaffold design to test its sensitivity to the loading condition definition. A first simulation was run assuming 50% higher loading values (compression force and bending moment) and a second one with 50% lower loading values. These changes could reflect uncertainties in the loading conditions really applying at the defect position, as well as varying behaviors (e.g. un-loading of the injured limb) after the surgery.

The MBBR simulations of the optimal titanium scaffold with 50% higher or lower loading conditions showed a rather low sensitivity to the loading conditions, as large amounts of regenerated bone and hardly no other tissue type were predicted in the scaffold pores after 24 weeks (Supplementary Figure 1). However, in both cases the regenerated bone volume fraction was lower: 90% for the higher loads and 79% for the reduced loads. In the latter case, more resorption zones were predicted in the callus and explained the lower healing outcome.


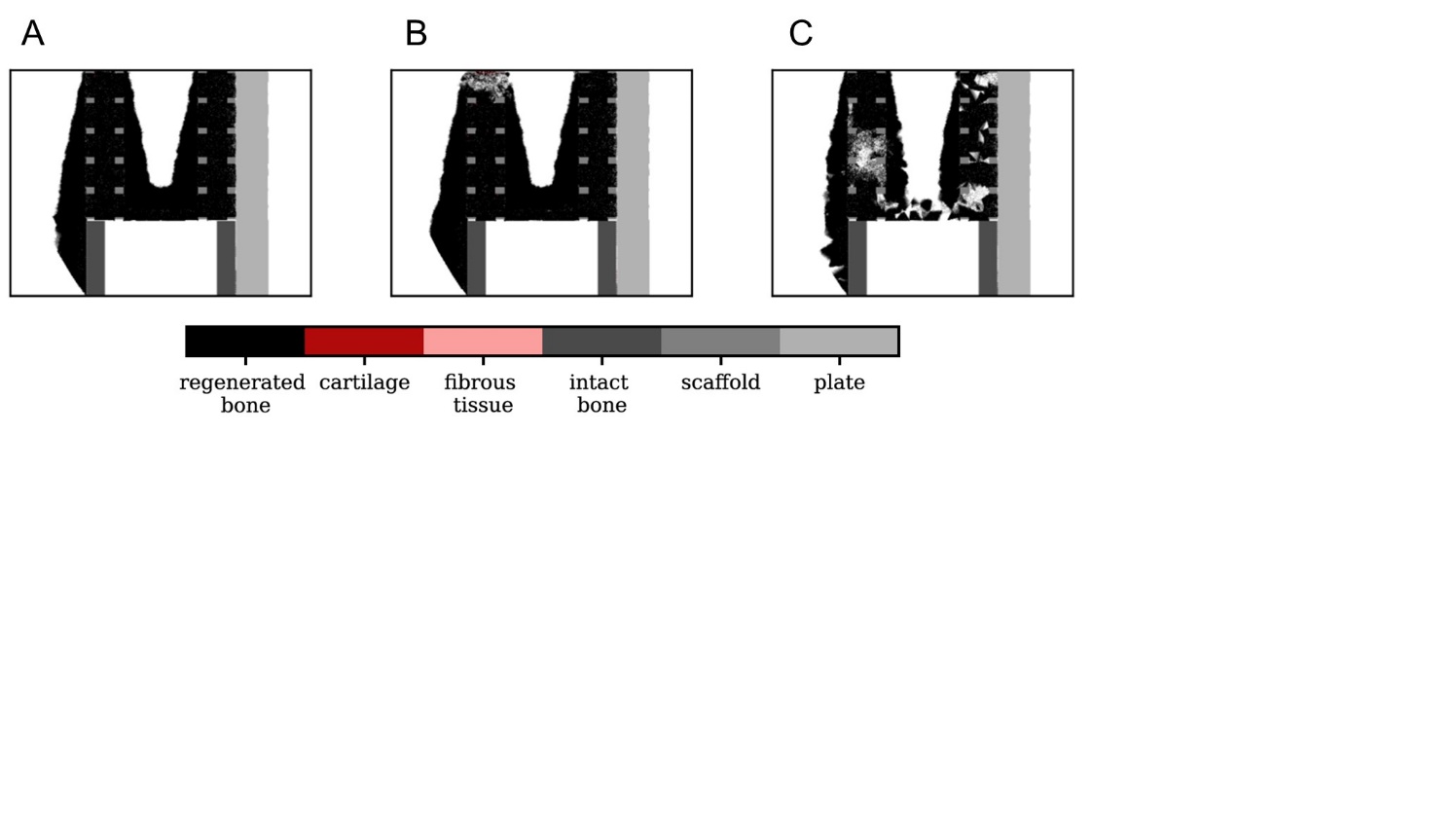


**Supplementary Figure 1.** 24-week histology predictions for the optimal titanium scaffold design under varying loading conditions: (A) baseline loading, used for the optimization; (B) loading values +50%; (C) loading values -50%.
